# Supplementary material for: A Microsatellite Genotyping-Based Genetic Study of Interspecific Hybridization between the Red and Sika Deer in the Western Czech Republic
Source: Animals (Basel). 2021 Jun 7;11(6):1701. doi: 10.3390/ani11061701 (PMC8229882; doi:10.3390/ani11061701)
Supplement: Supplementary file 1 [file animals-11-01701-s001.zip › Figure S1.pdf]

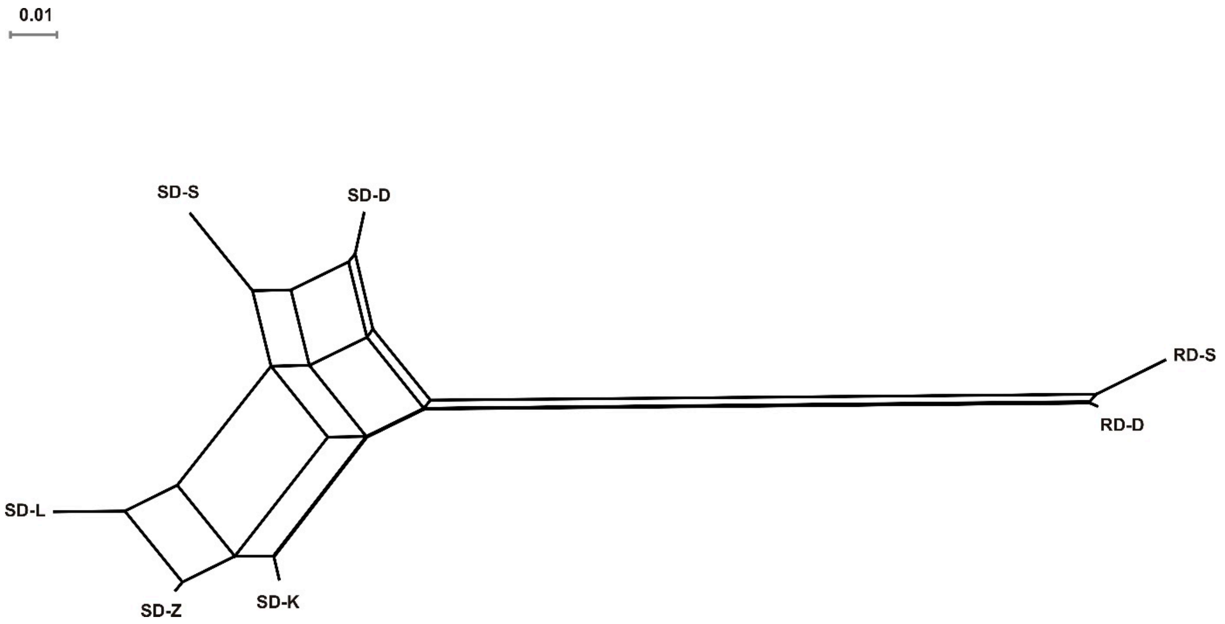

Figure S1. The NeighborNet dendrogram constructed from the Reynolds' D_R distances between the seven red and sika deer sampling populations (*n* = 123).

Population code: RD-D (Red deer Doupovské hory), RD-S (Red deer Slavkovský les), SD-D (Sika deer Doupovské hory), SD-S (Sika deer Slavkovský les), SD-K (Sika deer Kladská), SD-L (Sika deer Lány), and SD-Z (Sika deer Žlutice).
