# Supplementary material for: A Microsatellite Genotyping-Based Genetic Study of Interspecific Hybridization between the Red and Sika Deer in the Western Czech Republic
Source: Animals (Basel). 2021 Jun 7;11(6):1701. doi: 10.3390/ani11061701 (PMC8229882; doi:10.3390/ani11061701)
Supplement: Supplementary file 1 [file animals-11-01701-s001.zip › Table S1.pdf]

Table S1. The overall population statistics with/without hybrids.

**Population with hybrids**	***n***	**Loci typed**	**H_E ± SD**	**H_O ± SD**	**NA ± SD**
Red deer	63	11	0.8415 ± 0.0180	0.7305 ± 0.0171	13.00 ± 2.83
Sika deer	60	11	0.6809 ± 0.0327	0.6386 ± 0.0189	7.64 ± 2.20
**Population without hybrids**					
Red deer	54	11	0.8340 ± 0.0194	0.7168 ± 0.0187	12.18 ± 2.93
Sika deer	56	11	0.6680 ± 0.0343	0.6382 ± 0.0196	6.55 ± 1.92

H_E = expected heterozygosity; H_O = observed heterozygosity; NA = No of Alleles; SD = standard deviation
