# Supplementary material for: A Microsatellite Genotyping-Based Genetic Study of Interspecific Hybridization between the Red and Sika Deer in the Western Czech Republic
Source: Animals (Basel). 2021 Jun 7;11(6):1701. doi: 10.3390/ani11061701 (PMC8229882; doi:10.3390/ani11061701)
Supplement: Supplementary file 1 [file animals-11-01701-s001.zip › Table S2.pdf]

Table S2. The *p*-values for the genotypic disequilibrium between the pairwise loci within each species (obtained with FSTAT 2.9.4). The adjusted *p*-value for the 5% nominal level of the Bonferroni correction is 0.000909.

Pairwise loci	Red Deer	Sika Deer
OarFCB5 X T156	0.67909	0.64273
OarFCB5 X T26	0.14909	0.17091
OarFCB5 X BM888	0.12545	0.13727
OarFCB5 X RM188	0.62636	0.60909
OarFCB5 X RT1	0.22636	0.25545
OarFCB5 X T501	0.56636	0.59364
OarFCB5 X RT13	0.00727	0.01000
OarFCB5 X T193	0.97455	0.98000
OarFCB5 X BM1818	0.99818	0.99727
OarFCB5 X ETH225	0.78273	0.77909
T156 X T26	0.09364	0.09545
T156 X BM888	0.21182	0.19545
T156 X RM188	0.50545	0.54000
T156 X RT1	0.05273	0.06273
T156 X T501	0.05182	0.04909
T156 X RT13	0.35091	0.36818
T156 X T193	0.32818	0.32091
T156 X BM1818	0.98000	0.97182
T156 X ETH225	0.03455	0.02727
T26 X BM888	0.01636	0.02182
T26 X RM188	0.19909	0.16909
T26 X RT1	0.02182	0.01545
T26 X T501	0.27182	0.29636
T26 X RT13	0.01909	0.01091
T26 X T193	0.49182	0.47909
T26 X BM1818	0.55818	0.57727
T26 X ETH225	0.34909	0.33273
BM888 X RM188	0.23636	0.21818
BM888 X RT1	0.04818	0.04273
BM888 X T501	0.01364	0.00636
BM888 X RT13	0.14909	0.13091
BM888 X T193	0.06909	0.05727
BM888 X BM1818	0.58364	0.59091
BM888 X ETH225	0.94000	0.95455
RM188 X RT1	0.06455	0.08000
RM188 X T501	0.01182	0.00909
RM188 X RT13	0.09091	0.08455
RM188 X T193	0.88091	0.87545
RM188 X BM1818	0.47000	0.45636
RM188 X ETH225	0.27545	0.32545
RT1 X T501	0.48364	0.48182
RT1 X RT13	0.05091	0.04364

**Pairwise loci**	**Red Deer**	**Sika Deer**
RT1 X T193	0.11455	0.10727
RT1 X BM1818	0.96091	0.95545
RT1 X ETH225	0.37091	0.39364
T501 X RT13	0.00091*	0.00091*
T501 X T193	0.75364	0.77182
T501 X BM1818	0.58273	0.59364
T501 X ETH225	0.29000	0.29364
RT13 X T193	0.10909	0.12091
RT13 X BM1818	0.71364	0.69364
RT13 X ETH225	0.44818	0.43182
T193 X BM1818	0.27273	0.26364
T193 X ETH225	0.43818	0.43818
BM1818 X ETH225	0.71455	0.71000

* $p < 0.05$.
