# Supplementary material for: A Microsatellite Genotyping-Based Genetic Study of Interspecific Hybridization between the Red and Sika Deer in the Western Czech Republic
Source: Animals (Basel). 2021 Jun 7;11(6):1701. doi: 10.3390/ani11061701 (PMC8229882; doi:10.3390/ani11061701)
Supplement: Supplementary file 1 [file animals-11-01701-s001.zip › Table S3.pdf]

Table S3. The Nei´s D_A (below the diagonal) and the Reynolds' D_R genetic distances (above the diagonal) among the 7 populations studied.

	RD-D	SD-D	SD-K	SD-L	SD-S	SD-Z	RD-S
**RD-D**	0	0.1924	0.1964	0.2510	0.2237	0.2121	0.0207
**SD-D**	0.5921	0	0.0988	0.1086	0.0545	0.1184	0.2053
**SD-K**	0.6907	0.2372	0	0.0552	0.1034	0.0340	0.2137
**SD-L**	0.7276	0.2342	0.0686	0	0.1001	0.0370	0.2659
**SD-S**	0.6044	0.0957	0.2373	0.2006	0	0.1035	0.2431
**SD-Z**	0.7113	0.2718	0.0503	0.0513	0.2461	0	0.2285
**RD-S**	0.0958	0.6551	0.7596	0.7804	0.6849	0.7753	0

Population code: RD-D (Red deer Doupovské hory), RD-S (Red deer Slavkovský les), SD-D (Sika deer Doupovské hory), SD-S (Sika deer Slavkovský les), SD-K (Sika deer Kladská), SD-L (Sika deer Lány), and SD-Z (Sika deer Žlutice).
