# Supplementary material for: A Microsatellite Genotyping-Based Genetic Study of Interspecific Hybridization between the Red and Sika Deer in the Western Czech Republic
Source: Animals (Basel). 2021 Jun 7;11(6):1701. doi: 10.3390/ani11061701 (PMC8229882; doi:10.3390/ani11061701)
Supplement: Supplementary file 1 [file animals-11-01701-s001.zip › Table S4.pdf]

Table S4. Microsatellite data.

**Samples**	***OarFCB5***		***T156***		***T26***		***BM888***		***RM188***		***RT1***		***T501***		***RT13***		***T193***		***BM1818***		***ETH225***	
Animal_01	83	85	162	166	342	350	194	204	127	131	266	266	233	241	293	293	192	200	240	240	138	148
Animal_02	83	85	142	150	342	342	194	204	127	131	260	262	233	249	297	309	192	212	242	246	142	148
Animal_03	87	107	130	138	330	0	220	230	127	147	260	260	241	245	311	311	208	212	228	240	140	144
Animal_04	83	87	130	142	334	342	194	230	127	131	260	262	245	245	295	309	188	192	228	234	138	146
Animal_05	77	83	166	166	338	354	220	242	127	133	260	262	233	237	297	297	224	228	234	240	134	148
Animal_06	85	85	146	166	338	338	192	220	131	131	260	266	233	245	295	311	188	196	240	250	146	148
Animal_07	77	85	146	166	338	346	204	220	127	127	260	262	241	245	295	295	200	208	228	244	138	146
Animal_08	97	101	130	162	0	362	0	226	131	133	264	266	0	261	303	0	208	224	228	246	142	148
Animal_09	79	89	142	142	338	342	194	194	127	131	260	260	249	249	307	307	208	228	240	240	148	158
Animal_10	85	89	130	166	338	350	204	230	127	133	260	276	233	241	295	297	220	228	234	246	140	154
Animal_11	81	89	142	154	350	354	192	194	127	129	260	260	261	261	293	299	200	200	232	240	148	180
Animal_12	81	89	142	162	346	362	174	192	131	131	262	266	261	261	303	303	200	208	240	240	138	162
Animal_13	77	85	154	162	358	358	194	228	129	129	258	262	233	257	293	293	192	200	240	240	146	148
Animal_14	77	85	138	150	330	342	192	194	125	125	262	268	233	233	303	303	212	224	242	250	138	142
Animal_15	81	85	142	162	334	350	194	196	127	133	260	260	237	269	293	293	200	228	228	242	144	148
Animal_16	79	81	130	142	350	350	196	196	133	133	260	260	233	237	289	293	188	200	228	240	134	180
Animal_17	87	87	138	142	362	362	192	196	125	125	260	268	233	253	289	303	200	212	232	244	138	138
Animal_18	77	85	142	146	358	362	174	194	129	131	260	268	233	257	299	303	184	200	232	242	146	148
Animal_19	87	89	150	162	330	342	192	238	127	127	260	262	233	261	307	323	184	196	232	250	148	156
Animal_20	85	89	138	138	350	358	192	192	129	129	260	262	233	261	293	307	192	200	234	0	156	156
Animal_21	81	89	154	162	322	362	192	196	135	135	256	268	233	233	307	307	188	212	232	242	144	148
Animal_22	79	87	146	146	314	358	192	192	129	129	256	260	233	233	303	303	168	188	240	250	138	138
Animal_23	87	89	150	162	330	358	192	234	127	127	260	268	233	261	289	289	216	228	240	240	134	154
Animal_24	89	93	138	166	330	350	194	194	127	127	260	260	233	245	293	311	180	200	240	240	138	142
Animal_25	87	93	150	178	338	362	174	190	127	133	250	260	237	261	323	323	188	196	232	242	152	152
Animal_26	77	87	138	138	342	354	192	192	135	135	260	268	233	233	303	323	168	192	234	246	148	152
Animal_27	79	89	138	138	362	362	192	192	127	127	260	268	261	261	303	303	196	196	240	250	138	142
Animal_28	79	85	142	178	346	350	192	192	127	129	262	268	253	257	289	303	188	192	240	240	138	158
Animal_29	77	85	142	162	346	362	192	192	127	129	260	262	233	269	293	303	188	200	228	242	146	160
Animal_30	85	87	138	150	338	0	192	192	125	125	260	262	233	253	289	323	212	220	232	242	138	142
Animal_31	77	89	166	166	330	346	174	0	129	129	260	268	257	261	301	301	172	188	234	240	138	142
Animal_32	77	101	130	178	342	358	194	238	129	129	260	268	253	261	303	303	200	212	240	240	138	146
Animal_33	85	89	166	174	338	346	190	194	127	127	260	262	265	265	293	303	196	220	240	250	134	146
Animal_34	79	79	130	138	0	362	194	0	125	127	262	268	237	0	291	291	188	196	244	250	156	180
Animal_35	85	89	130	162	342	362	174	194	127	131	256	260	233	265	307	307	212	220	232	242	138	146
Animal_36	83	83	142	142	338	358	194	240	127	129	260	280	233	233	289	323	188	196	234	246	146	158
Animal_37	87	89	138	162	330	334	190	192	127	129	260	260	261	0	303	311	220	220	240	250	138	142
Animal_38	89	89	146	162	334	354	190	242	127	129	260	264	233	233	303	303	220	0	240	240	146	154
Animal_39	77	81	138	154	346	350	192	194	131	131	260	262	261	261	289	303	208	224	234	240	146	146
Animal_40	77	89	138	170	358	370	192	192	127	129	260	268	233	0	301	301	208	212	240	242	142	146
Animal_41	85	85	150	154	346	358	204	242	127	127	266	266	233	265	297	323	212	220	232	240	146	146
Animal_42	77	85	150	166	358	358	190	192	131	131	268	270	233	237	325	0	188	200	232	242	138	146
Animal_43	87	95	150	150	358	362	192	196	123	131	260	272	245	261	303	307	180	200	240	240	138	156
Animal_44	79	89	162	166	358	358	174	174	127	135	260	260	257	257	311	323	204	208	232	234	148	148
Animal_45	81	83	150	150	346	346	174	224	129	129	258	260	233	253	303	311	228	0	234	250	138	148
Animal_46	87	87	130	142	334	350	194	224	127	129	268	268	233	233	289	301	228	228	240	250	148	154
Animal_47	89	107	130	142	350	354	192	192	127	137	260	260	237	253	293	293	192	216	232	240	138	138
Animal_48	81	85	130	162	354	354	194	232	129	129	260	280	253	265	289	293	228	228	232	240	142	154
Animal_49	85	89	142	142	0	350	194	216	127	135	260	260	237	261	307	307	192	196	240	242	146	154
Animal_50	79	87	150	162	346	350	190	194	127	127	260	260	233	269	285	285	192	192	234	240	138	154
Animal_51	79	85	130	162	350	350	192	216	129	129	260	262	233	269	285	325	208	224	234	250	138	138
Animal_52	79	79	142	166	314	358	192	222	127	131	260	260	233	261	293	293	212	228	240	240	146	146
Animal_53	87	89	134	0	334	346	174	196	129	133	260	262	0	257	293	293	188	192	246	250	142	160
Animal_54	79	101	126	146	342	358	190	192	129	139	262	268	249	253	289	289	220	228	228	244	142	158
Animal_55	83	87	162	178	346	350	192	230	127	127	256	256	233	257	289	303	220	224	232	232	138	138

Samples	*OarFCB5*		*T156*		*T26*		*BM888*		*RM188*		*RT1*		*T501*		*RT13*		*T193*		*BM1818*		*ETH225*	
Animal_56	81	85	138	178	350	362	194	196	129	139	260	280	233	233	303	323	196	0	244	250	138	138
Animal_57	89	89	142	178	350	362	174	222	129	129	260	268	233	233	303	323	188	192	244	244	138	138
Animal_58	85	85	130	154	334	362	192	194	125	125	260	260	0	257	293	313	208	212	246	250	138	138
Animal_59	85	87	130	162	350	362	174	174	129	131	260	280	233	257	285	293	200	220	228	240	148	156
Animal_60	89	93	146	208	338	366	194	194	129	129	260	260	257	257	329	329	208	228	234	250	138	154
Animal_61	79	87	162	162	354	358	190	232	129	137	260	262	237	237	303	311	216	220	228	232	138	154
Animal_62	87	89	130	162	330	354	190	192	125	129	256	280	0	273	289	293	220	228	244	244	138	156
Animal_63	77	77	142	162	350	362	194	194	133	133	260	260	237	257	303	303	208	212	234	242	142	156
Animal_64	85	87	142	150	342	350	200	204	145	155	264	272	241	245	297	301	260	260	240	240	144	144
Animal_65	85	89	142	150	350	350	200	204	145	145	270	272	245	245	307	307	260	336	240	240	142	142
Animal_66	85	99	150	182	350	354	200	200	145	155	266	272	245	245	307	307	260	320	240	254	144	148
Animal_67	85	85	174	182	350	354	200	200	0	185	264	272	245	249	297	297	320	332	240	254	142	158
Animal_68	85	85	150	166	350	354	200	200	145	165	264	270	241	245	301	301	260	276	240	240	142	166
Animal_69	85	85	166	166	338	342	200	200	145	165	264	270	245	245	297	307	260	260	242	268	142	144
Animal_70	85	85	166	182	342	354	190	200	145	145	264	264	245	249	297	301	276	276	240	254	142	142
Animal_71	85	85	166	170	338	350	190	202	147	157	266	266	229	257	303	309	260	366	242	254	142	144
Animal_72	85	85	150	174	350	354	198	210	147	147	266	266	235	261	299	299	260	320	254	254	144	144
Animal_73	79	85	146	150	0	366	200	212	147	155	270	282	237	259	313	0	260	260	240	240	142	144
Animal_74	79	87	166	182	336	348	200	212	145	145	264	264	243	245	297	309	316	320	240	240	144	144
Animal_75	85	89	140	174	340	348	200	204	145	163	264	266	245	249	297	303	260	320	242	254	142	142
Animal_76	85	87	144	182	336	348	200	204	147	155	266	272	245	249	297	297	260	320	254	254	144	148
Animal_77	85	87	166	182	340	348	200	212	147	155	264	270	243	249	303	309	260	320	240	242	142	144
Animal_78	85	87	170	182	336	340	200	204	145	155	264	270	245	245	297	303	260	276	240	240	142	144
Animal_79	79	87	140	144	340	348	200	200	145	153	264	272	245	245	297	307	260	260	254	0	142	144
Animal_80	85	89	170	182	340	348	204	210	145	153	264	266	243	245	303	309	260	276	240	254	144	144
Animal_81	85	89	166	182	348	348	204	210	147	155	264	264	245	249	307	309	260	336	242	268	142	144
Animal_82	81	85	148	174	336	340	200	204	145	147	266	270	245	249	303	307	320	332	268	0	142	144
Animal_83	85	89	166	170	348	348	200	200	145	145	264	270	245	245	301	307	320	332	240	240	142	144
Animal_84	85	85	144	170	340	348	198	200	147	155	270	270	245	245	301	301	316	320	242	254	144	144
Animal_85	85	85	166	0	340	340	200	200	145	163	264	264	245	245	297	297	260	336	254	268	142	142
Animal_86	85	87	148	166	336	348	204	210	145	145	266	272	243	245	297	303	260	276	240	254	142	144
Animal_87	85	85	140	174	340	340	200	204	145	153	264	266	243	245	297	307	260	320	242	268	142	144
Animal_88	85	87	148	148	348	348	200	210	145	155	272	272	245	245	297	307	260	336	240	240	144	144
Animal_89	85	85	148	174	336	348	204	204	145	145	264	264	245	249	297	297	316	320	254	254	142	144
Animal_90	85	87	148	166	340	348	200	200	145	153	264	264	245	245	297	297	260	260	242	268	142	144
Animal_91	85	87	0	166	340	348	200	200	145	145	264	264	243	245	297	301	260	260	240	240	144	144
Animal_92	85	85	166	182	340	340	200	204	145	145	264	266	245	245	301	307	260	276	254	254	142	144
Animal_93	81	85	170	182	336	348	190	200	145	145	264	272	243	245	297	303	320	336	242	268	142	144
Animal_94	85	85	148	170	348	348	204	210	0	163	264	272	243	249	297	297	260	260	240	240	144	144
Animal_95	85	85	140	148	340	348	204	204	145	163	264	264	245	249	297	297	260	320	240	254	144	144
Animal_96	85	85	166	174	0	350	190	202	147	147	266	266	243	0	305	309	260	332	254	268	142	144
Animal_97	85	85	166	170	334	354	198	210	147	147	266	266	245	245	0	309	260	316	240	254	144	144
Animal_98	85	85	142	166	342	354	200	200	145	165	264	264	245	245	301	307	260	320	242	254	142	142
Animal_99	85	85	150	166	338	342	190	200	145	145	264	264	241	245	297	307	320	336	254	254	142	144
Animal_100	85	99	150	150	342	354	200	204	145	145	264	264	241	249	301	307	260	332	240	240	142	142
Animal_101	85	85	166	170	354	354	200	204	145	155	264	264	245	245	297	307	260	260	254	254	142	144
Animal_102	85	85	142	182	342	342	200	200	0	155	264	266	241	249	303	303	260	320	240	240	144	144
Animal_103	85	89	166	166	338	342	200	200	141	0	262	264	245	245	297	307	320	336	254	0	142	144
Animal_104	85	89	150	166	350	366	200	204	145	157	264	270	245	245	301	307	260	260	240	254	142	144
Animal_105	81	89	166	166	362	370	198	198	141	145	264	270	245	245	307	307	260	336	254	254	142	144
Animal_106	81	81	166	174	350	354	200	200	145	157	264	270	245	245	301	301	260	336	240	240	144	144
Animal_107	81	85	170	174	350	350	198	200	145	165	264	264	245	245	299	301	276	316	240	254	142	142
Animal_108	81	85	150	174	342	350	200	204	145	145	262	264	245	245	297	301	276	320	240	242	144	144
Animal_109	81	93	166	166	342	350	198	204	141	145	264	270	245	245	301	307	260	320	254	254	144	144
Animal_110	81	81	166	166	342	350	200	200	145	0	262	264	245	245	301	301	260	260	240	254	142	144
Animal_111	89	93	166	166	342	342	200	200	147	147	264	270	245	245	301	301	316	320	240	240	142	144
Animal_112	85	85	150	174	350	350	210	210	145	155	264	264	245	245	297	307	320	332	240	254	144	144

Samples	*OarFCB5*		*T156*		*T26*		*BM888*		*RM188*		*RT1*		*T501*		*RT13*		*T193*		*BM1818*		*ETH225*	
Animal_113	85	85	150	166	338	342	200	200	145	155	264	264	241	245	297	297	260	320	240	240	144	144
Animal_114	81	85	150	166	342	354	200	200	155	0	264	264	245	245	297	303	260	320	240	254	142	144
Animal_115	87	87	166	170	342	354	190	200	145	145	264	264	245	249	297	297	260	260	240	254	142	144
Animal_116	79	79	166	166	336	336	198	212	145	145	264	264	243	245	297	309	316	320	254	268	142	144
Animal_117	85	87	140	144	336	348	198	200	145	163	264	266	245	245	297	303	260	320	254	254	142	144
Animal_118	85	87	140	174	340	340	200	212	145	155	266	272	243	249	297	303	260	336	240	240	144	144
Animal_119	85	85	166	182	340	348	204	210	145	155	264	266	245	249	297	297	260	276	240	242	142	142
Animal_120	81	85	148	166	336	348	200	204	145	153	264	272	243	245	297	307	260	260	240	240	144	144
Animal_121	85	89	166	182	340	340	200	212	145	163	264	272	245	245	297	301	316	320	242	242	144	144
Animal_122	85	89	166	170	348	348	204	210	145	153	264	264	243	245	303	309	260	276	254	254	142	144
Animal_123	85	87	148	148	340	348	204	210	145	163	264	266	243	245	297	303	320	332	254	268	144	144
